# Population- and Species-Level Variation in Near- and Mid-infrared Radiation in Birds: A Preliminary Analysis

**DOI:** 10.1093/iob/obag006

**Published:** 2026-02-28

**Authors:** T Lee, M Barrett, L Pilon, A J Shultz, T McGlynn

**Affiliations:** Mechanical and Aerospace Engineering Department, Henry Samueli School of Engineering and Applied Science, University of California, Los Angeles, CA 90095, USA; Department of Biology, California State University Dominguez Hills, Carson, CA 90747, USA; Department of Biology, Indiana University Indianapolis, Indianapolis, IN 46202, USA; Mechanical and Aerospace Engineering Department, Henry Samueli School of Engineering and Applied Science, University of California, Los Angeles, CA 90095, USA; Department of Ornithology, Natural History Museum of Los Angeles County, Los Angeles, CA 90007, USA; Department of Biology, California State University Dominguez Hills, Carson, CA 90747, USA; Department of Ornithology, Natural History Museum of Los Angeles County, Los Angeles, CA 90007, USA

## Abstract

Animal coloration has diverse functions, such as camouflage, communication, thermoregulation, and protection from UV damage and more, and can be shaped by environmental selective pressures. Some climatic selective pressures are strong enough to produce consistent patterns in many species across large-scale geographic gradients, leading to the creation of macrophysiological rules such as Gloger’s rule, which predicts that endothermic populations in hot, humid areas will be visibly darker than those in cool, dry areas, and the thermal melanism hypothesis, which predicts that ectothermic animals will be visibly darker in cooler areas. While these rules often capture trends in animal absorptance in the visible spectrum, wavelengths of visible light are not the only relevant wavelengths to an animal’s energy budget: solar radiation extends beyond the visible spectrum [0.4–0.7 μm] into the near-infrared; thus, thermal pressures may result in changes in surface reflectance characteristics beyond the visible [e.g., 0.7–2.5 μm] in birds. Further, heat exchange with the environment extends into the mid-infrared (MIR), including heat loss through the atmospheric transparency window [8–14 μm]. It is unknown whether animal absorptance in the NIR or emittance in MIR might also follow macrophysiological rules, as seen in the visible spectrum, such as more absorptance of NIR and less emittance of MIR in cooler areas for ectotherms under the thermal melanism hypothesis. Here, we examine both UV-NIR absorptance and MIR emittance in five species of birds: the Great Horned Owl, Northern Bobwhite, Steller’s Jay, Song Sparrow, and Common Raven. We show that NIR absorptance varies by species and population, corresponding to their habitat and thermoregulatory strategies. MIR emittance, in contrast, was stable across species and populations but differed slightly across populations of Northern Bobwhites. We conclude by highlighting the importance of considering the full spectrum from UV to MIR in research on animal adaptation. Further consideration of infrared radiation is necessary for a complete view of animals’ phenotypic diversity and possible responses to thermal challenge.

## Version Francaise

La coloration animale a des fonctions diverses telles que le camouflage, la communication, la thermorégulation, la protection contre les dommages dus aux UV et bien d’autres. Elle peut être façonnée par des pressions sélectives environnementales. Certaines pressions sélectives climatiques sont suffisamment fortes pour produire des motifs cohérents chez de nombreuses espèces le long de gradients géographiques à grande échelle, conduisant à la création de règles macrophysiologiques. Par exemple, la règle de Gloger prédit que les populations endothermes dans des zones chaudes et humides seront visiblement plus sombres que celles des zones fraîches et sèches, et l’hypothèse du mélanisme thermique, qui prédit que les animaux ectothermes seront visiblement plus sombres dans les zones plus fraîches. Bien que ces règles rendent souvent compte des tendances de l’absorbance des animaux dans le spectre visible, les longueurs d’onde de la lumière visible ne sont pas les seules pertinentes pour le budget énergétique d’un animal : le rayonnement solaire s’étend au-delà du spectre visible [0.4–0.7 μm] dans le proche infrarouge (IR). Ainsi, les pressions thermiques peuvent entraîner des modifications de la réflectance au-delà du visible [e.g., 0.7–2.5 μm] chez les oiseaux. En outre, les échanges de chaleur avec l’environnement s’étendent à l’infrarouge moyen, y compris la perte de chaleur MIR à travers la fenêtre de transmission atmosphérique [8–14 μm]. On ignore si l’absorbance animale dans le proche IR ou l’émittance dans l’IR moyen pourraient également suivre des règles macrophysiologiques, comme observé dans le spectre visible, comme par exemple, une plus grande absorbance dans le proche IR et une moindre émittance dans l’IR moyen dans les zones plus froides chez les ectothermes selon l’hypothèse du mélanisme thermique. Ici, nous examinons à la fois l’absorbance de l’UV au proche infrarouge et l’émittance dans l’infrarouge moyen chez cinq espèces d’oiseaux : le Grand-duc d’Amérique, le Colin de Virginie, le Geai de Steller, le Bruant chanteur et le Grand Corbeau. Nous montrons que l’absorbance dans l’infrarouge proche varie selon les espèces et les populations, en fonction de leur habitat et de leurs stratégies de thermorégulation. L’émittance dans l’infrarouge moyen, en revanche, est restée stable entre les espèces et les populations, mais différait légèrement entre les populations de Colin de Virginie. Nous concluons en soulignant l’importance de considérer l’ensemble du spectre de l’UV à l’infrarouge moyen dans la recherche sur l’adaptation animale. Une prise en compte plus approfondie du rayonnement infrarouge est nécessaire pour obtenir une vision complète de la diversité phénotypique des animaux et de leurs réponses possibles aux contraintes thermiques.

## Introduction

External coloration is one mechanism by which animals adapt to various selective pressures in their environment. In the visible [VIS; 0.4–0.7 μm] spectrum, this complex phenotypic trait has diverse functions, such as camouflage, intraspecific communication, parasite deterrence, thermoregulation, and more (Cuthill et al. 2017; Stuart-Fox et al. 2017; Delhey et al. 2019; Delhey 2020; Trigo and Mota 2015). Where absorptance is expected to have a thermoregulatory function, climate variation across large-scale climate gradients may be correlated with animal absorptance (Chown et al. 2004). In the visible spectrum, variance in animal absorptance (e.g., coloration) can be described by macrophysiological rules like Gloger’s rule, which posits that endothermic animals will be darker in humid and warm areas due to the increased deposition of melanin in their surface structures (Delhey 2019). This macroecological pattern is often observed across latitudinal gradients, with darker populations of the same species found in wetter and warmer environments closer to the equator (e.g., Caro 2005; Hogstad et al. 2009). Another macroecological pattern related to visible coloration is the thermal melanism hypothesis or Bogert’s rule, which has generally been explored in ectotherms and predicts darker colored individuals in cooler environments where absorptance of solar radiation is considered key to maintaining the animals’ metabolism (Clusella-Trullas et al. 2008; Martínez-Freiría et al. 2020).

Both Gloger’s rule and the thermal melanism hypothesis deal with an animal’s expected absorptance in the visible spectrum; however, absorption of other wavelengths of light may also play a role in animals’ energy balances and thermoregulation. About 55% of incoming solar energy falls within the near infrared [NIR, wavelengths from 701 to 2500 nm; (Stuart-Fox et al. 2017)]. Thus, adaptations that yield changes in absorptance in the NIR could play a strong role in thermoregulation without some of the confounds that limit adaptation in the visible spectrum (NIR absorptance cannot be seen by conspecifics or predators; Stuart-Fox et al. 2017). Indeed, interspecific variation in NIR absorptance has been found to correlate strongly with climate variation in insects and birds (Medina et al. 2018; Munro et al. 2019; Kang et al. 2021; Mason et al. 2023; though see Porter et al. 2023; Wang et al. 2023). Even within species, reduced NIR absorptance can correlate with hotter microclimate specialization (e.g., in bees; Barrett and O’Donnell 2023). Despite relatively extensive cross-species comparisons, few studies have assessed if there may be variation in NIR absorptance among populations of birds.

In addition to the reflection of solar irradiance in the UV-NIR, animals can also exchange longwave radiation with their environment as another mechanism of thermoregulation. Substantial heat emittance in the mid-infrared (MIR, typically, 7.5–14 um; Howell et al. 2015) can allow animals to cool down by using space as a heat sink, due to the highly transmissive atmosphere across this spectral range (Taylor and Yates 1957). Alternatively, low emittance in the MIR wavelengths would allow animals to retain heat. Insects found in hot environments have increased emittance as a method of remaining cool, irrespective of other climate variables such as precipitation and humidity (Krishna et al. 2020, 2021). Variation in MIR emittance can even be specific to a body region: for instance, the Saharan silver ants, known for foraging in an extremely hot microclimate, have very high emittance on their dorsal surface to lose heat via the atmospheric transmission window and low emittance on the ventral surface to avoid heat exchange with the very hot desert ground (Shi et al. 2015). Variation in MIR emittance has never been studied in birds inter- or intraspecifically.

In this study, we test for interspecific and intraspecific variation in NIR absorptance and MIR emittance. We selected three or four, geographically diverse populations from five species of birds with wide latitudinal distributions. Four species have plumage variation corresponding to Gloger’s rule [Great Horned Owl *Bubo virginianus* (Ostrow et al. 2023), Northern Bobwhite *Colinus virginianus* (Johnsgard and Jones 1988), Song Sparrow *Melospiza melodia* (Burtt and Ichida 2004), and Steller’s Jay *Cyanocitta stelleri* (Wiebe 1995)]; one species does not have plumage variation corresponding to Gloger’s rule (Common Raven *Corvus corax*). We also selected species that are known to be primarily open-habitat species (Northern Bobwhite, Common Raven), closed-habitat species (Song Sparrow, Steller’s Jay), or nocturnal species (Great Horned Owl), to facilitate comparisons among species with different solar exposure. We used spectrometry to assess UV-NIR absorptance and MIR emittance of the dorsal surface of birds (specifically, the mantle) of three to six birds from each population for each species and assessed both interspecific and intraspecific variation. As we measured whole birds with bodies that are not transparent in UV-MIR radiation (even if single layers of feathers might be), we were able to assume 0 transmittance through our specimens (e.g., 1 minus reflectance will equal absorptance in the UV-MIR). We assessed patterns in visible “coloration” differences first by assessing bird-visible (300–700 nm) and human-visible (400–700 nm) absorptance coefficients. We then further analyzed any trends in UV-NIR (300–2500 nm) absorptance and MIR emittance across species and populations.

## Materials and methods

### Sampling

We choose populations based on the availability of high-quality specimens in the Natural History Museum of Los Angeles County collection, where at least three samples per population were required. For each species except Song Sparrows, we selected three populations with differences in environmental conditions, largely corresponding with latitudinal variation. From each population, we selected three adult specimens to account for individual variation (Supplemental Table 1 for specimen age, sex, etc. information), resulting in an *n* = 9 per species (Supplemental Fig. 1 for representative photos of each species). For Song Sparrows, (Supplemental Table 1), we sampled six adult specimens from four populations, resulting in an *n* = 24 for this species. Due to sample availability and the amount of time needed to collect these data per sample (as well as the potential for damage to sensitive instruments from natural specimens that are quite dirty compared to most materials used with the machines), larger sample sizes were not possible for all species. Where possible given sample availability, we chose specimens of the same sex for analysis (e.g., in Northern Bobwhites), but this was often not possible. Although these that are not strongly sexually visually dichromatic on the mantle where measurements would be taken, it remains a possibility worth future testing to determine if there is strong sexual dichromatism in the NIR or MIR.

**Table 1:** Terms and definitions

Term	Definition
UV	Ultraviolet wavelengths of the electromagnetic spectrum, 300–400 nm.
VIS	Visible wavelengths of the electromagnetic spectrum, 400–700 nm.
NIR	Near infrared wavelengths of the electromagnetic spectrum, 0.7–2.5 μm.
MIR	Mid Infrared wavelengths of the electromagnetic spectrum, 2.5–20 μm.
Shortwave radiation	Radiative exchange associated with shorter wavelengths that are emitted by the sun or reflected and absorbed by a surface wavelength range includes UV to NIR.
Normal-hemispherical	The method used to measure the reflection of light when a beam of light is incident on a surface, then measured with a detector that captures light in a hemisphere.
Normal–normal	The method used to measure the reflection of light when a beam of light is incident on a surface, then measured with a detector that captures light in a small solid angle close to the incident beam.
Absorption	The process of a material taking in electromagnetic radiation (light) and converting it to internal energy.
Absorptance	The fraction of incident radiation that is absorbed by a surface or material; ranges from 0 to 1.
Blackbody	The theoretical perfect absorber (maximum absorptance of incident radiation at all wavelengths and directions) and the perfect emitter (maximum emittance at a given temperature at any wavelength in any direction).
Emittance	The fraction of blackbody radiation that is emitted by a surface or material for a given temperature; ranges from 0 to 1. Bird body temperature typically around 37°C will emit mostly in the MIR spectrum.
Reflectance	The fraction of incident radiation that is reflected by a surface or material; ranges from 0 to 1. Coloration of a bird depends on its reflectance in the UV-VIS spectrum.

#### Climate data acquisition

Climate data was obtained from the NOAA National Centers for Environmental Information’s (NCEI) Annual Climate Maps tool, using the most recent data from the closest NOAA station to the specimen. Only temperature and precipitation data was collected, as reported in each station’s annual report, for annual average temperature, the average maximum and minimum temperatures (per day over a year), extreme maximum and minimum temperature of that was recorded during the year, total liquid precipitation of the year, and highest liquid precipitation content recorded that year. No spatial or temporal resolution was explicitly reported by the NOAA stations. Data was accessed from the interactive map at https://www.ncei.noaa.gov/maps/annual/on November 8, 2024 (and July 10, 2025 for sparrows).

### Spectrometry

#### Normal-normal reflectance probe

We measured normal–normal reflectance in the spectral range of 300–700 nm using a UV–Vis spectrophotometer (Flame-S-UV–Vis, Ocean Optics), using a pulsed Xenon lamp (PX-2, Ocean Optics), and 400 μm reflectance probe (WS-1-SL, Ocean Optics) fitted with a modified rubber stopper to exclude all incident light. Reflectance spectra were collected relative to a spectralon diffuse white standard (WS-1-SL, Ocean Optics) with a 500 ms integration time, boxcar width of 5 and averaging 10 scans. To account for measurement uncertainty, we averaged three measurements on the mantle of each specimen. Most reflectance data in current literature uses normal–normal reflectance, which does not account for back-scattered photons (photons reflected not in the normal direction of the surface). Due to the complex morphology of bird feathers, we needed to use an integrating sphere to capture back-scattered photons from normally incident radiation.

#### UV-Vis spectrometer

The spectral normal-hemispherical reflectance (Table 1) *R*_nh,λ_ of the dorsal surface in the spectral range of 300–1100 nm was measured using a UV–Vis spectrometer (Evolution TM 201 UV–Visible Spectrometer, Thermo Scientific) fitted with an integrating sphere accessory (DRA-EV-600, Thermo Scientific). Integration time was set to 0.2 s with spectral increments of 1 nm. The spectral normal-hemispherical reflectance *R*_nh,λ_ was estimated according to


(1) \begin{equation*}{R}_{nh,\lambda } = \frac{{{S}_{nh,\lambda } - {D}_{nh,\lambda }}}{{{B}_{nh,\lambda } - {D}_{nh,\lambda }}}{R}_{std,\lambda }.\end{equation*}

The spectral normal-hemispherical reflectance signal *S*_nh,λ_ measured by the UV–Vis spectrometer was corrected by subtracting the dark signal *D*_nh,λ_ measured by blocking any light from entering the detector. The baseline spectral normal-hemispherical reflectance measurement *B*_nh,λ_ was determined using a calibrated specular reflection standard mirror (NIST certified STAN-SSH, Ocean Optics) with known standard normal-hemispherical reflectance *R*_std,λ_. To account for measurement uncertainty, we averaged three measurements on the mantle of each specimen.

#### FTIR

A nitrogen-purged Fourier transform infrared (FTIR) spectrometer (Nicolet TM iS50, Thermo Scientific Fischer, USA) equipped with an integrating sphere (Upward IntegratIRTM, PIKE Technologies, USA) was used to measure the spectral normal-hemispherical reflectance *R*_nh,λ_ of the dorsal surface in the NIR and MIR. A liquid-nitrogen cooled mercury–cadmium–telluride (MCT) detector and a KBr beamsplitter were used to measure *R*_nh,λ_ in the range between 2 and 20 μm, and an InGaAs detector and a Calcium fluoride (CaF_2_) were used to measure *R*_nh,λ_ in the range between 1 and 2.5 μm. Similar to the UV–Vis spectrometer, the spectral normal-hemispherical reflectance *R*_nh,λ_ was estimated according to


(2) \begin{equation*}{R}_{nh,\lambda } = \frac{{{S}_{nh,\lambda } - {D}_{nh,\lambda }}}{{{B}_{nh,\lambda } - {D}_{nh,\lambda }}}.\end{equation*}

The spectral normal-hemispherical reflectance signal *S*_nh,λ_ measured by the FTIR was corrected by subtracting the dark signal *D*_nh,λ_ measured by blocking any light from entering the detector. The baseline spectral normal-hemispherical reflectance measurement *B*_nh,λ_ was determined using a gold reflectance standard (Thermo Fisher). To account for measurement uncertainty, we averaged 600 scans on the mantle of each specimen. For each bird species, the reflectance, dark, and baseline signals for a given spectrometer and detector ensemble was collected on the same day. For the owl and raven species, measurements on the FTIR were supported by an optical stand so that the region between the feathers could be measured consistently.

#### Post-collection data cleaning/manipulation information

Some of the spectral ranges measured by the spectrophotometers overlapped and to calculate total emittance or total absorptance, the data was condensed to have one reflectance value per wavelength. For the region between 1 and 1.1 μm, data sets collected from the UV–Vis and the InGaAs detectors were approximately linear in this region so a linear interpolation was used to merge both data sets. For the region between 2 and 2.5 μm, the MCT and InGaAs detectors captured similar reflectance curves indicative of chemical absorption peaks by N–H bonds in keratin (Miyamae et al. 2007). To preserve the shape of these curves, the MCT data was used for data processing so that the 2–20 μm could be kept continuously without any manipulation of data (Supplemental Fig. 2, e.g., in sparrows).

The bird feathers are assumed to be dielectric materials that have optically rough surfaces so the emittance of the bird feathers are independent of direction (i.e., a diffuse emitter). We also assume that the dorsal surface is not transparent for the wavelength range of UV-MIR. Using Kirchoff’s law, the spectral normal-hemispherical emittance ε_nh,λ_ and spectral normal-hemispherical absorptance α_nh,λ_ is expressed as (Howell et al. 2015)


(3) \begin{equation*}{\varepsilon }_{nh,\lambda } = {\alpha }_{nh,\lambda } = 1 - {R}_{nh,\lambda }.\end{equation*}

Then, the total normal-hemispherical emittance ε_nh_ is defined as (Howell et al. 2015)


(4) \begin{eqnarray*}
{\varepsilon }_{nh} = \frac{{\int_{0}^{\infty }{{{\varepsilon }_{nh,\lambda }{I}_{b,\lambda }({T}_s)d\lambda }}}}{{\int_{0}^{\infty }{{{I}_{b,\lambda }({T}_s)d\lambda }}}},
\end{eqnarray*}

where *I_b_*_,λ_(*T_s_*) is the spectral Planck’s blackbody intensity at surface temperature, *T_s_*, taken as 313 K (Simpson 1922), and integrals in the numerator and denominator were truncated with bounds of 0.3 and 20 μm for the available spectral data obtained by our instruments. This truncation considers 78% of full blackbody emittance energy.

The solar absorptance αs was calculated according to


(5) \begin{eqnarray*}{\alpha }_s = \frac{{\int_{0}^{\infty }{{{\alpha }_{nh,\lambda }{G}_{sol,\lambda }d\lambda }}}}{{\int_{0}^{\infty }{{{G}_{sol,\lambda }d\lambda }}}},\end{eqnarray*}

where *G*_sol,λ_ is the ASTM G-173 Spectra (Howell et al. 2015). Here also, the integrals were truncated with bounds of 0.3 and 20 μm for the available spectral data obtained by our instruments. This truncation considers 97% of the total blackbody radiation *I_b_*(*T_s_*) and solar radiation through AM1.5G.

Additionally, we used the reflectance data to calculate the average brilliance of the birds (Trigo and Mota 2015) using the formula shown below:


(6) \begin{eqnarray*}
\overline {{R}_b} = \frac{{\sum\nolimits_{300\,{\mathrm{mm}}}^{700\,{\mathrm{nm}}} {{R}_{\lambda ,i}} }}{n},
\end{eqnarray*}

where *R*_λ_,*_i_* is the reflectance data at a wavelength between 300 and 700 nm and *n* is the number of integer wavelengths between 300 and 700 nm.

### Statistical analyses

All data are available on Dryad: https://doi.org/10.5061/dryad.fxpnvx13b.

We used GraphPad Prism v. 9.3.1 (GraphPad Software 2023) to analyze all data. We used Shapiro–Wilk normality tests to assess normal distribution of the data. We used a one-way Analysis of Variance (ANOVA) followed by Tukey’s Multiple Comparisons Test for normally distributed data or a Kruskal–Wallis test with Dunn’s Multiple Comparisons Test for data that were not normally distributed. These methods were used to assess species-level differences in emittance and population-level differences in absorptance and emittance within each species. For ravens, we also tested differences between the two named subspecies by combining the data for two populations (for all other species all populations come from different named subspecies). Here, we used a Welch’s *t*-test to test for differences among subspecies in absorptance, to account for unequal variance, and an unpaired *t*-test to test for differences between subspecies in emittance. Normal–normal reflectance data was used for cross-species comparisons, and for all intraspecific comparisons with owls and ravens (as normal-hemispherical data could not be gathered for these species due to size-related limitations). Normal-hemispherical reflectance data was used for intraspecific comparisons in bobwhites, jays, and sparrows. Linear regressions were used to assess the relationship between an UV-NIR reflectance and mean annual temperature data, and the relationship between VIS and NIR absorptance coefficients for bobwhites. For all tests, alpha was set to 0.05.

## Results

### Species-level absorptance and emittance

Normal–normal reflectance data demonstrated significant variation in mean UV-NIR absorptance (α; 300–2500 nm) across species (Supplemental Table 2, Supplemental Fig. 3, Fig, 1; Kruskal–Wallis, K-W = 34.88, *P* < 0.0001). Owls had a significantly lower mean absorptance than ravens and sparrows (Dunn’s MCT: owl-raven: *Z* = 5.38, *P* < 0.0001; owl-sparrow: *Z* = 3.27, *P* = 0.0109). Bobwhites also had lower mean absorptance than ravens (*Z* = 4.38, *P* = 0.0001). There was no difference in the normal-hemispherical reflectance/absorptance of bobwhites, sparrows, or jays (Kruskal–Wallis; K-W: 4.68, *P* = 0.0966).

**Fig. 1:**
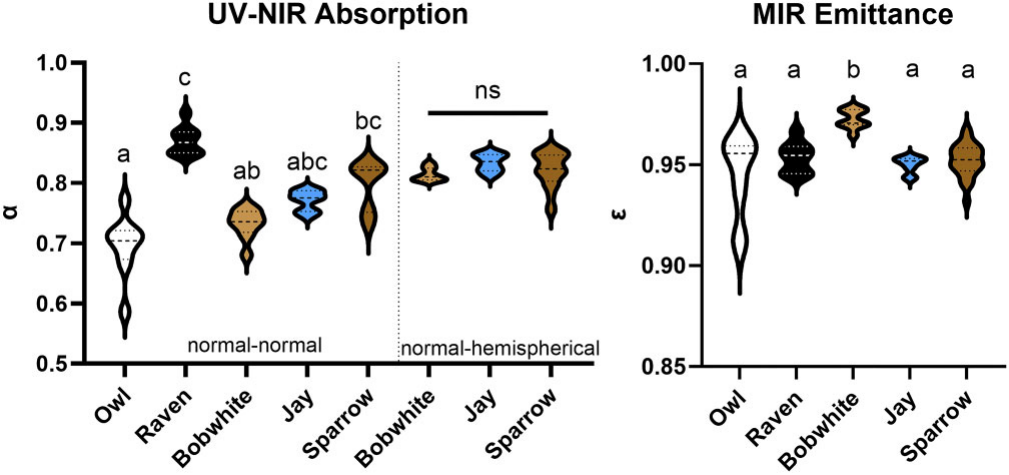
Variation in (left) solar absorptance and (right) emittance coefficients for each species. Normal–normal data demonstrated that there was significant variation in mean solar absorptance (α) across species (Supplemental Table 2; *P* < 0.0001); there was no difference in normal-hemispherical absorptance across species (*P* = 0.09). Bobwhites had significantly higher emittance than all other species (*P* < 0.05). Letters indicate statistically significant differences among species. ns, not significant.

**Table 2:** Differences in human-visible (VIS; 400–700 nm) absorptance across populations (*n* = 3 birds/population, except for sparrows [*n* = 6 birds/population]).

Species	Subspecies*	Populations (average temp)	α—mean ± SD (*n*)	Statistical information
Owl**	*occidentalis* *pallescens* *pacificus*	Wyoming, USA/Canada (5°C) California, USA (desert) (15.5°C) California, USA (coast) (18.4°C)	0.86 ± 0.04 (3) 0.82 ± 0.05 (3) 0.89 ± 0.009 (3)	*F* = 1.86; *df* = 2; *P* = 0.24
Bobwhite	*mexicanus* *insignis* *floridanus*	Iowa, USA (10.6°C) Chiapas, MX (19.3°C) Florida, USA (24.3°C)	0.97 ± 0.009 (3) 0.97 ± 0.003 (3) 0.97 ± 0.006 (3)	*F* = 0.01; *df* = 2; *P* = 0.99
Raven**	*principalis* *sinatus* (A) *sinatus* (B)	Alaska, USA (8.4°C) California, USA (16.4°C) Sinaloa, MX/BajaCa, MX (24.2°C)	0.99 ± 0.007 (3) 0.99 ± 0.01 (3) 0.99 ± 0.004 (3)	*F* = 0.47; *df* = 2; *P* = 0.65
Jay	*stellari^a^* *frontalis^b^* *diademata^b^*	Alaska, USA (8.2°C) California, USA (11.8°C) Chihuahua, MX (16.4°C)	0.99 ± 0.004 (3) 0.97 ± 0.006 (3) 0.96 ± 0.004 (3)	*F* = 27.35; *df* = 2; ***P* = 0.001**
Sparrow	*caurina^a^* *cooperi^ab^* *merrilli^b^* *saltonis^c^*	Alaska, USA (8.3°C) S. California, USA (coastal) (16.7°C) N. California, USA (16.4°C) S. California, USA (desert) (18.4°C)	0.98 ± 0.007 (6) 0.98 ± 0.009 (6) 0.97 ± 0.003 (6) 0.95 ± 0.01 (6)	*F* = 19.95; *df* = 3; ***P* < 0.0001**

*Letters indicate statistically significant differences using Tukey’s posthoc test (following ANOVA) or Dunn’s posthoc test (following Kruskal–Wallis). Significant p-values are bolded.

**Absorptance coefficients for owl and raven are normal–normal; the other species are normal-hemispherical.

There was also variation in mean total hemispherical MIR emittance (ε) across species (Supplemental Table 2, Supplemental Fig. 3, Fig. 1; Kruskal–Wallis; K-W = 23.11, *P* < 0.0001). Bobwhites had significantly higher emittance than all species (Dunn’s MCT; bobwhite-owl: *Z* = 3.75, *P* = 0.0018; bobwhite-raven: *Z* = 3.24, *P* = 0.012; bobwhite-jay: *Z* = 4.19, *P* = 0.0003; bobwhite-sparrow: *Z* = 4.16, *P* = 0.0003). All other species were not significantly different (Fig. 1; *Z* < 0.95, *P* > 0.99).

In both cases, owls had the greatest range of absorptance and emittance, and thus the most variation among individuals. The range of absorptance in UV-NIR across species was much greater than the range of emittance in MIR (UV-NIR range of means: 0.70–0.87; MIR: 0.94–0.97; Supplemental Table 2).

### Population-level trends in human-visible (400–700 nm) absorptance

Gloger’s rule and the thermal melanism hypothesis are based on differences in reflectivity across the human-visible spectrum (VIS; 400–700 nm), not on the total solar spectrum (300–2500 nm). To assess if there were population-level trends in any of our focal species consistent with Gloger’s rule or the thermal melanism hypothesis, we tested for human-visible changes in coloration (and bird-visible results [300–700 nm] are in Supplemental Table 3). Results were also analyzed as “average brilliance” (where the reflectance at each wavelength from 400 to 700 nm is averaged, without accounting for incoming solar energy as in the absorptance coefficient analysis), a technique commonly used for birds (Supplemental Table 4).

**Table 3:** Differences in solar (UV-NIR) absorptance across populations (*n* = 3 birds/population, except for sparrows [*n* = 6 birds/population]).

Species	Subspecies*	Populations (mean temp)	α—mean ± SD (*n*)	Statistical information
Owl**	*occidentalis* *pallescens* *pacificus*	Wyoming, USA/Canada (5°C) California, USA (desert) (15.5°C) California, USA (coastal) (18.4°C)	0.70 ± 0.03 (3) 0.66 ± 0.07 (3) 0.72 ± 0.04 (3)	KW = 1.87; *P* = 0.44
Bobwhite	*mexicanus^c^* *insignis^b^* *floridanus^ab^*	Iowa, USA (10.6°C) Chiapas, MX (19.3°C) Florida, USA (24.3°C)	0.83 ± 0.003 (3) 0.81 ± 0.004 (3) 0.80 ± 0.001 (3)	*F* = 29.38; *df* = 2; ***P* = 0.0008**
Raven**	*principalis* *sinatus* (A)*** sinatus (B)	Alaska, USA (8.4°C) California, USA (16.4°C) Sinaloa, MX, Baja CA (24.2°C)	0.85 ± 0.003 (3) 0.89 ± 0.02 (3) 0.87 ± 0.02 (3)	*F* = 3.82, *df* = 2, *P* = 0.09
Jay	*stellari* *frontalis* *diademata*	Alaska, USA (8.2°C) California, USA (11.8°C) Chihuahua, MX (16.4°C)	0.85 ± 0.003 (3) 0.82 ± 0.01 (3) 0.83 ± 0.01 (3)	*F* = 4.26; *df* = 2; *P* = 0.07
Sparrow	*caurina^a^* *cooperi^a^* *merrilli^b^* *saltonis^c^*	Alaska, USA (8.3°C) S. California, USA (coastal) (16.7°C) N. California, USA (16.4°C) S. California, USA (desert) (18.4°C)	0.85 ± 0.008 (6) 0.84 ± 0.01 (6) 0.82 ± 0.004 (6) 0.78 ± 0.02 (6)	*F* = 35.09; *df* = 3; ***P* < 0.0001**

*Letters indicate statistically significant differences using Tukey’s posthoc test (Bobwites) or Dunnett’s T3 (sparrows). Significant p-values are bolded.

**Absorptance coefficients for owl and raven are normal–normal; the other species are normal-hemispherical.

***Ravens, when analyzed by subspecies, differed significantly (Welch’s *t*-test: *t* = 3.56, *df* = 5.33, *P* = 0.0145), with *C. sinatus* having increased absorptance compared to *C. principalis*.

**Table 4:** Differences in emittance across populations of each species (*n* = 3 birds/population, except for sparrows [*n* = 6 birds/population]).

Species	Subspecies	Populations (mean temp)	ε—mean ± SD (*n*)	Statistical information
Owl	*occidentalis* *pallescens* *pacificus*	Wyoming, USA/Canada (5°C) California, USA (desert) (15.5°C) California, USA (coastal) (18.4°C)	0.94 ± 0.03 (3) 0.94 ± 0.02 (3) 0.95 ± 0.01 (3)	*F* = 0.24; *df* = 2; *P* = 0.79
Bobwhite	*mexicanus^b^* *insignis^a^* *floridanus^ab^*	Iowa, USA (10.6°C) Chiapas, MX (19.3°C) Florida, USA (24.3°C)	0.98 ± 0.01 (3) 0.97 ± 0.001 (3) 0.97 ± 0.01 (3)	*W* = 98.23; *df* = 2; *P* = **0.0008**
Raven**	*principalis* *sinatus* (A) *sinatus* (B)	Alaska, USA (8.4°C) California, USA (16.4°C) Sinaloa, MX, Baja CA (24.2°C)	0.96 ± 0.01 (3) 0.95 ± 0.008 (3) 0.95 ± 0.006 (3)	*F* = 1.14, *df* = 2, *P* = 0.28
Jay	*stellari* *frontalis* *diademata*	Alaska, USA (8.2°C) California, USA (11.8°C) Chihuahua, MX (16.4°C)	0.95 ± 0.004 (3) 0.95 ± 0.003 (3) 0.95 ± 0.001 (3)	*F* = 2.73; *df* = 2; *P* = 0.14
Sparrow	*caurina* *cooperi* *merrilli* *saltonis*	Alaska, USA (8.3°C) S. California, USA (coastal) (16.7°C) N. California, USA (16.4°C) S. California, USA (desert) (18.4°C)	0.96 ± 0.009 (6) 0.95 ± 0.007 (6) 0.95 ± 0.009 (6) 0.95 ± 0.006 (6)	*F* = 2.07; *df* = 3; *P* = 0.14

*Letters indicate statistically significant differences using Dunnett’s T3 MCT. Significant p-values are bolded.

**No difference between subspecies: Unpaired *t*-test, *t* = 1.15, *df* = 7, *P* = 0.29.

**Fig. 2:**
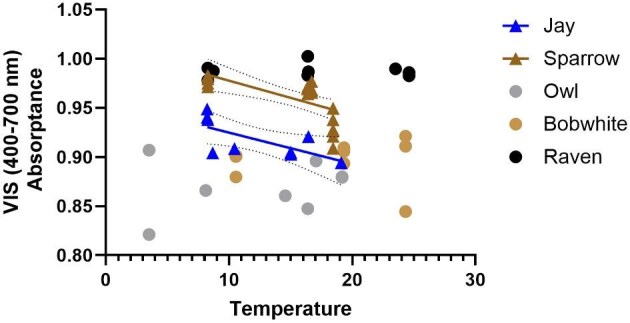
VIS absorptance negatively correlates with a population’s mean annual temperature in jays (*n* = 3 populations, 9 individuals) and sparrows (*n* = 4 populations, 24 individuals). Linear regression for jays (*R*^2^ = 0.47, *F* = 6.10, *df* = 7, *P* = 0.0428): [VIS absorptance] = −0.003 [temperature] + 0.96. Linear regression for sparrows (*R*^2^ = 0.40, *F* = 12.93, *df* = 19, *P* = 0.0019): [VIS absorptance] = −0.003 [temperature] + 1.01). Solid lines indicate the line of best fit, dots indicate the 95% CI.

There were significant differences among populations/subspecies in VIS absorptance for jays and sparrows (Table 2; normal-hemispherical); in both cases, VIS absorptance negatively correlated with the population’s mean annual temperature (Fig. 2; linear regressions, both *P* < 0.043).

Jays from Mexico and California did not differ in their VIS absorptance (Tukey’s MCT: *q* = 1.79, *df* = 6, *P* = 0.46), while jays from Alaska had increased VIS absorptance (visibly darker coloration) compared to both other populations (Alaska–Mexico: *q* = 9.82, *df* = 6, *P* = 0.0011; Alaska–California: *q* = 8.03, *df* = 6, *P* = 0.0031). Sparrows populations also differed in VIS absorptance (*F* = 19.95, *P* < 0.0001; normal-hemispherical). VIS absorptance was lowest (lighter coloration) in sparrows from the desert region of southern California compared to all other populations and highest in sparrows from Alaska (all *P* < 0.033), while birds from northern California and the coastal regions of southern California had intermediate VIS absorptance (Table 2).

### Population-level trends in total solar (UV-NIR, 300–2500 nm) absorptance

**Fig. 3:**
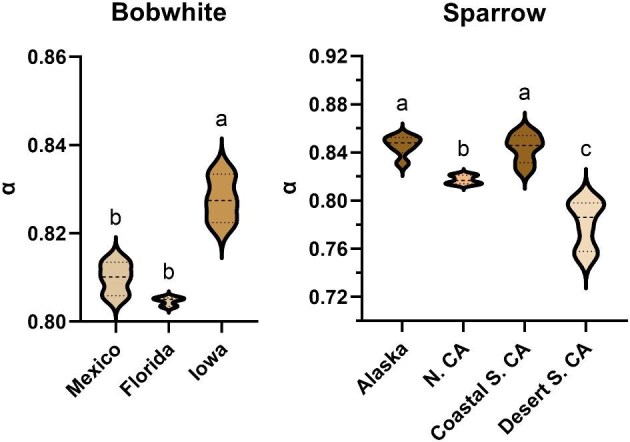
Population-level differences in solar absorptance coefficients for (left) bobwhites and (right) sparrows. Bobwhites from Iowa had increased absorptance compared to those from Mexico and Florida, though comparisons are limited by the small sample size (*n* = 3 birds/population). Sparrows from the desert region of California had lower absorptance coefficients than those from Alaska and coastal S. California or N. California; those from N. California had lower absorptance coefficients than those from Alaska or coastal S. California (*n* = 6 birds/population). ns, not significant; ** = *P* < 0.01; *** = *P* < 0.001; **** = *P* < 0.0001.

There were significant differences among populations/subspecies in UV-NIR absorptance for bobwhites and sparrows (ANOVA, Brown-Forsythe ANOVA, respectively; Table 3; normal-hemispherical), though results of population-level analyses outside of sparrows should be interpreted with caution due to low power from our sample size. Bobwhites from Mexico and Florida did not differ in their UV-NIR absorptance coefficients (Tukey’s MCT: *q* = 2.32, *df* = 6, *P* = 0.30), while bobwhites from Iowa had increased UV-NIR absorptance (darker coloration) compared to both other populations (Fig. 3; Iowa–Mexico: *q* = 8.01, *df* = 6, *P* = 0.0031; Florida–Iowa: *q* = 10.33, *df* = 6, *P* = 0.0008). Interestingly, bobwhites did not differ in absorptance coefficients in the VIS specifically (Table 2) but did when considering the entire UV-NIR range (Table 3). Linear regression analysis showed that VIS (400–700 nm) absorptance did not predict NIR (700–2500 nm) absorptance, suggesting a decoupling of reflectance in these wavelength bands (*F* = 3.12, *df* = 7, *P* = 0.12).

Sparrows from Alaska and the coastal region of S. California did not differ in their UV-NIR absorptance coefficients (*t* = 0.39, *df* = 8.89, *P* > 0.99); all other populations differed (all *P* < 0.03), with sparrows from the desert regions of S. California having the lowest UV-NIR absorptance coefficient (lighter coloration) (Fig. 3). UV-NIR absorptance was significantly and negatively correlated with mean annual temperature in bobwhites (*R*^2^ = 0.89, *F* = 54.84, *df* = 7, *P* = 0.0001, [UV-NIR absorptance] = −0.002 [temperature] + 0.85) and sparrows (*R*^2^ = 0.42, *F* = 13.69, *df* = 19, *P* = 0.0015, [UV-NIR absorptance] = −0.005 [temperature] + 0.89).

Ravens did not show differences among populations (ANOVA; Table 3), but subspecies showed differences in absorptance (Welch’s *t*-test: *t* = 3.56, *df* = 5.33, *P* = 0.0145), where the mean absorptance of *C. corax sinatus* was significantly higher (darker coloration) than *C. corax principalis* (Supplemental Fig. 4). In this case, the subspecies from the warmer locations had *increased* mean absorptance compared to the subspecies from the cooler location (the opposite pattern as seen in sparrows and bobwhites).

### Population-level trends in MIR emittance

**Fig. 4:**
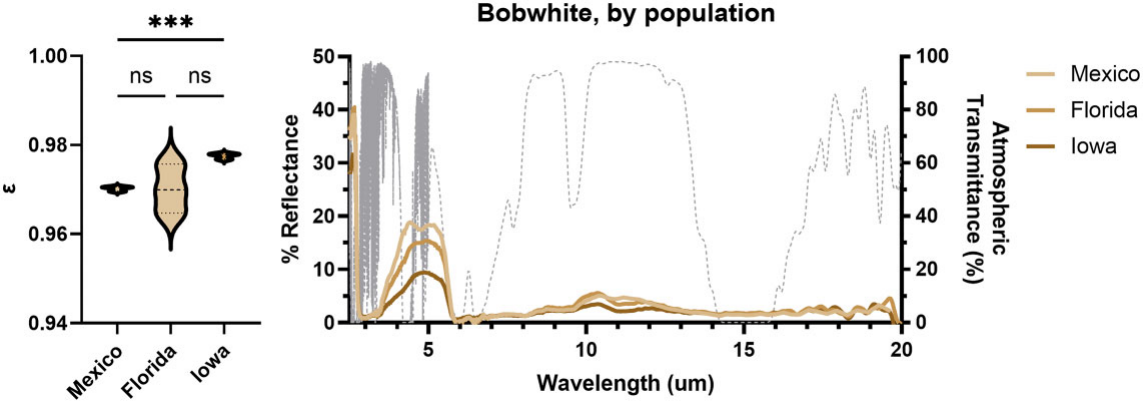
Population-level differences in (left) emittance coefficients and (right) MIR reflectance spectra in bobwhites. Bobwhites from Iowa and Florida, and Florida and Mexico, did not differ in their emittance coefficients, while bobwhites from Iowa had significantly higher emittance than those from Mexico, though comparisons are limited by the small sample size (*n* = 3 birds/population). The gray dashed line shows atmospheric transmission (%). ns, not significant; *** = *P* < 0.001.

There were significant differences among populations/subspecies in emittance for bobwhites (ANOVA; Table 4) but not owls, sparrows, ravens, or jays. Bobwhites from Iowa and Florida, and Florida and Mexico, did not differ in MIR emittance (Fig. 4; Dunnett’s T3; Mexico–Florida: *t* = 0.03, *df* = 2.04, *P* > 0.99; Florida–Iowa, *t* = 2.32, *df* = 2.06, *P* = 0.29), while bobwhites from Iowa (the coolest population) had significantly higher emittance than those from Mexico (*t* = 15.25, *df* = 3.86, *P* = 0.0003). However, in the atmospheric transmission window (∼7.5–14 μm), differences in reflectance values for bobwhites were not pronounced.

## Discussion

We found variation in UV-NIR absorptance and MIR emittance across the bird species we sampled as well as across populations within some species. For instance, owls have significantly lower mean absorptance in the UV-NIR than several diurnal species, such as ravens and sparrows, as well as a greater range of reflectance values across the UV-NIR compared to the other species. This increased range of values may reflect relaxed selective pressure on the reflectance of solar radiation across the entire solar spectrum in this species, as owls are nocturnal. Alternatively, owls are large and patterned (at least, in the VIS spectrum), with many layers of feathers; these factors may contribute to greater variance in measurement consistency among individuals. Reduced selective pressure on reflectance as a thermal adaptation may also explain why owl populations did not differ from one another in either UV-NIR absorptance or MIR emittance, despite being known to follow Gloger’s rule (which could be expected to drive at least some variation in UV-NIR absorptance, as it is inclusive of the VIS spectrum; Ostrow et al. 2023). We also found that bobwhites had notably higher MIR emittance than all other tested species, though interspecific differences in emittance were all within 3% for our tested species.

Although our sample sizes are limited (outside of the song sparrows) and thus our power is low for intraspecific analyses, our data still indicate there may be intraspecific differences across bird populations in mean absorptance in the UV-NIR. In both bobwhites and sparrows, UV-NIR absorptance was increased in cooler regions/at higher latitudes, and there was a similar but ultimately non-significant trend in jays. This suggests that these population-level differences in UV-NIR reflectance may be consistent with a thermally driven hypothesis, wherein hotter environments may be populated by lighter colored (e.g., more reflective) individuals to reduce heat stress. Although not yet studied intraspecifically, similar results were found in interspecific bird pigmentation analyses (Galván et al. 2018) and other UV-NIR analyses in birds (Medina et al. 2018), as well as UV-NIR studies of gastropods and butterflies (Munro et al. 2019; Franklin et al. 2022).

Prior research has demonstrated that there are trade-offs between camouflage and thermoregulation when considering VIS spectrum absorptance. For example, brighter horned larks in a given environment had increased cooling but worse camouflage in the VIS spectrum (Mason et al. 2023). While we found that sparrow populations differed whether considering just the VIS or the entire UV-NIR, populations of bobwhites only differed in absorptance when the entire UV-NIR spectrum (and not only the VIS) was considered. It is possible that decoupling the degree of variation in the VIS from the NIR allows bobwhites to achieve “the best of both worlds”—remaining well-camouflaged in the VIS while reducing their heat load through changes in NIR/total solar absorptance; our data support this decoupling, showing that VIS (400–700 nm) reflectance did not predict NIR (700–2500 nm) reflectance. Bobwhites are open habitat grassland species that experience high levels of solar radiation depending on their habitat structure, potentially driving these stronger differences across populations.

Ravens are also open habitat nesters, but the species showed high UV-NIR absorptance. When analyzed by subspecies/population, ravens in warmer climates actually had *increased* absorptance compared to those from cooler areas (the opposite of the pattern observed for sparrows and bobwhites). Although initially it would seem as though darker plumage must always increase body temperature through increased absorptance of solar energy, studies in some darkly colored birds have shown that darker plumage can sometimes offer a thermoregulatory advantage in environments with high solar load and high wind velocities, such as those produced by active flight (Wolf and Walsberg 2000). Lighter plumage can reflect more radiation, but also allows increased penetration of that radiation into the plumage, potentially resulting in increased heating of the skin; darker plumage absorbs more solar radiation, but this heat is kept at the surface of the animal where it can more easily be dispersed convectively (Walsberg 1982). Studies in the brown-necked raven showed that black plumage heated up more than light plumage at the animal’s surface, but skin temperature remained the same (Marder 1973). At wind velocities greater than 5.5 m/s, pigeons with black plumage had reduced solar heat loads compared to those with white plumage (Walsberg et al. 1978; Wolf and Walsberg 2000). Accordingly, ravens in warmer climates with increased solar load may still benefit from the increased UV-NIR absorptance of their dorsal feathers.

While again acknowledging our limited sample sizes which mean that caution should be used in interpreting our results, we found that MIR emittance only differed by population in bobwhites, with greater dorsal emittance found in higher latitude populations with cooler climates. This is the first demonstration of intraspecific, population-level differences in MIR emittance in animals. The direction of the population differences in MIR emittance in bobwhites are surprising, as greater MIR emittance on the dorsal surface would be predicted in *hotter* climates to offload heat through the atmospheric transmission window (see, in insects: Shi et al. 2015; Krishna et al. 2021). There are several possible explanations to explain why we found the opposite pattern of our prediction. First, cloud coverage and atmospheric moisture might be better predictors of population-level variance in MIR emittance than temperature or latitude, as these variables impact the strength of the sky as a radiative sink (see Hardy and Stoll 1954; Nobel 1991). Thus, these variables could be more important than air temperature in determining how the animal’s energy balance is impacted by MIR emittance (though see Krishna et al. 2021, where precipitation was not linked to MIR emissivity in butterflies). Second, it is possible that emittance varies based on the “side” of the feather measured (e.g., emittance of MIR radiation from the bird’s body going into the environment may differ from emittance of external MIR radiation entering the bird’s body). Third, differences in MIR emittance may be so slight among populations as to be negligible to the animals’ heat budget, resulting in largely no patterns among populations with occasional statistically significant “noise”—particularly when there is lower power in a study, such as ours. More focused sampling of bobwhites based on these preliminary findings would help rule in, or out, hypothesis three. Given that differences in emittance were only 1% across populations, and that longwave radiative heat exchange generally represents a relatively smaller fraction of birds’ energy budgets (e.g., ∼8%, Ward et al. 1999; though see Léger and Larochelle 2006 under more naturalistic conditions), it seems plausible that if hypothesis two can be ruled out, hypothesis three is most likely in the species of birds we tested.

While conducting this study, we encountered some methodological constraints that deserve further attention in studies that focus on UV-NIR absorptance and MIR emittance in birds. For example, some birds have clearer VIS patterning on the dorsal surface than others (consider owls vs. ravens); accounting for this variance when using spectrometers that only assess a “spot” of a certain diameter can be challenging. Further, the size of birds can create challenges when attempting to obtain measurements—most spectrometers are designed for use with small material samples, not large specimens like many birds. In our study, this constrained our ability to obtain normal-hemispherical NIR absorptance for all species (which would be considered the more ecologically relevant measure). Owls and ravens were too large to fit in the spectrometer, and only normal–normal absorptance could be obtained. Further, analyzing feathers separated from birds did not provide accurate results (presumably due to a lack of appropriate layering), with a three-fold difference in measured dorsal reflectance for an intact Song Sparrow compared to a single one of its feathers (Supplemental Fig. 5). Given the time-intensive nature of sampling and the sensitivity of the machines to damage from dusty/dirty samples, as well as the spotty availability of specimens with the same sex, age, season of collection, and/or preparation method, conducting a study with appropriate power to test all of these variables would be challenging. However, even with our limited sample size, we were able to discern some interesting differences across populations that suggest further research in this area is warranted.

For species where both normal–normal and normal-hemispherical data could be obtained, means differed by 3–8% between the measurements, suggesting that study results could differ based on the ability to obtain normal-hemispherical vs. normal–normal data on whole birds (Table 1). Feather morphology may magnify the different relationships between normal-hemispherical and normal–normal data across species, with some feathers likely to increase the amount of diffuse reflectance off the sample—thus increasing the relevance of normal-hemispherical data. Finally, further research is needed to understand how specimen preparation could impact MIR measurements: for instance, year of collection, age of specimen, method of filling, or how specimens are stored could impact MIR measurements and therefore the methodology itself deserves further attention in birds.

Our data reinforce prior findings that UV-NIR absorptance varies among birds. In addition, our data suggests that intraspecific variation may correspond to habitat characteristics, which should be further explored with larger sample sizes and more species. We found that birds in warmer climates, or at lower latitudes, tended to have reduced UV-NIR absorptance. This suggests that warmer climates and increased solar load may drive the evolution of reduced UV-NIR absorptance in birds’ dorsal feathers as a thermal adaptation. Further, our results suggest there may be detectable differences in MIR emittance across populations in some species. However, these differences in MIR emittance are quite slight in the species we tested (not greater than 5% in either interspecific or intraspecific variation), representing very little of the animal’s overall thermal budget, and thus suggesting that thermal selective pressure on MIR emittance is not as great in endotherms as has been found in some ectotherms (variation in emissivity of 40% in butterflies from different climates; Krishna et al. 2021). Still, selection can operate on very small margins, so our data suggests that variation in MIR emittance should be further studied in birds. Finally, alongside the other methodological and ecological factors already mentioned as deserving further attention, an expanded study that considered additional life history characteristics such as migration may also be of value, as some nocturnal migratory birds could be expected to have a greater reliance on longwave radiative heat exchange through the atmospheric transmission window (Léger and Larochelle 2006).

## Supplementary Material

obag006_Supplemental_Files

## Data Availability

All data are available on Dryad: https://doi.org/10.5061/dryad.fxpnvx13b.
