# Supplementary material for: Population- and Species-Level Variation in Near- and Mid-infrared Radiation in Birds: A Preliminary Analysis
Source: Integr Org Biol. 2026 Feb 28;8(1):obag006. doi: 10.1093/iob/obag006 (PMC13048275; doi:10.1093/iob/obag006)
Supplement: obag006_Supplemental_Files [file obag006_supplemental_files.zip › (French) Abstract.docx]

**VERSION FRANCAISE**

La coloration animale a des fonctions diverses telles que le camouflage, la communication, la thermorégulation, la protection contre les dommages dus aux UV et bien d’autres. Elle peut être façonnée par des pressions sélectives environnementales. Certaines pressions sélectives climatiques sont suffisamment fortes pour produire des motifs cohérents chez de nombreuses espèces le long de gradients géographiques à grande échelle, conduisant à la création de règles macrophysiologiques. Par exemple, la règle de Gloger prédit que les populations endothermes dans des zones chaudes et humides seront visiblement plus sombres que celles des zones fraîches et sèches, et l’hypothèse du mélanisme thermique, qui prédit que les animaux ectothermes seront visiblement plus sombres dans les zones plus fraîches. Bien que ces règles rendent souvent compte des tendances de l’absorbance des animaux dans le spectre visible, les longueurs d’onde de la lumière visible ne sont pas les seules pertinentes pour le budget énergétique d’un animal : le rayonnement solaire s’étend au-delà du spectre visible [0.4-0.7 μm] dans le proche infrarouge (IR). Ainsi, les pressions thermiques peuvent entraîner des modifications de la réflectance au-delà du visible [e.g., 0.7-2.5 μm] chez les oiseaux. En outre, les échanges de chaleur avec l’environnement s’étendent à l’infrarouge moyen, y compris la perte de chaleur MIR à travers la fenêtre de transmission atmosphérique [8 - 14 μm]. On ignore si l’absorbance animale dans le proche IR ou l’émittance dans l’IR moyen pourraient également suivre des règles macrophysiologiques, comme observé dans le spectre visible, comme par exemple, une plus grande absorbance dans le proche IR et une moindre émittance dans l’IR moyen dans les zones plus froides chez les ectothermes selon l’hypothèse du mélanisme thermique. Ici, nous examinons à la fois l’absorbance de l’UV au proche infrarouge et l’émittance dans l’infrarouge moyen chez cinq espèces d’oiseaux : le Grand-duc d’Amérique, le Colin de Virginie, le Geai de Steller, le Bruant chanteur et le Grand Corbeau. Nous montrons que l’absorbance dans l’infrarouge proche varie selon les espèces et les populations, en fonction de leur habitat et de leurs stratégies de thermorégulation. L’émittance dans l’infrarouge moyen, en revanche, est restée stable entre les espèces et les populations, mais différait légèrement entre les populations de Colin de Virginie. Nous concluons en soulignant l’importance de considérer l’ensemble du spectre de l’UV à l’infrarouge moyen dans la recherche sur l’adaptation animale. Une prise en compte plus approfondie du rayonnement infrarouge est nécessaire pour obtenir une vision complète de la diversité phénotypique des animaux et de leurs réponses possibles aux contraintes thermiques.
