# Supplementary material for: Population- and Species-Level Variation in Near- and Mid-infrared Radiation in Birds: A Preliminary Analysis
Source: Integr Org Biol. 2026 Feb 28;8(1):obag006. doi: 10.1093/iob/obag006 (PMC13048275; doi:10.1093/iob/obag006)
Supplement: obag006_Supplemental_Files [file obag006_supplemental_files.zip › Supp Table 1.docx]

**Supplemental Table 1. Catalog numbers and details of each specimen.**

| genus | species | subspecies | institution | catalog_number | sex | year | locality |
| --- | --- | --- | --- | --- | --- | --- | --- |
| Colinus | virginianus | floridanus | lacm | 5674 | m | 1894 | Orlando, FL, USA |
| Colinus | virginianus | floridanus | lacm | 5673 | m | 1894 | Orlando, FL, USA |
| Colinus | virginianus | floridanus | lacm | 5672 | m | 1894 | Orlando, FL, USA |
| Colinus | virginianus | insignis | lacm | 80966 | m | 1971 | Comitán, Chiapas, MX |
| Colinus | virginianus | insignis | lacm | 80969 | m | 1971 | Comitán, Chiapas, MX |
| Colinus | virginianus | insignis | lacm | 80970 | m | 1971 | Comitán, Chiapas, MX |
| Colinus | virginianus | mexicanus | lacm | 5665 | m | 1895 | Marshall Co., IA, USA |
| Colinus | virginianus | mexicanus | lacm | 5667 | m | 1895 | Marshall Co., IA, USA |
| Colinus | virginianus | mexicanus | lacm | 5670 | m | 1895 | Marshall Co., IA, USA |
| Bubo | virginianus | pallescens | lacm | 122863 | f | 2022 | Los Angeles Co., CA, USA |
| Bubo | virginianus | pallescens | lacm | 120717 | f | 2017 | Inyo Co., CA, USA |
| Bubo | virginianus | pallescens | lacm | 4070 | m | 1920 | California |
| Bubo | virginianus | pacificus | lacm | 104886 | f | 1990 | Los Angeles, CA, USA |
| Bubo | virginianus | pacificus | lacm | 110573 | m | 1998 | Los Angeles, CA, USA |
| Bubo | virginianus | pacificus | lacm | 123767 | m | 2021 | Los Angeles Co., CA, USA |
| Bubo | virginianus | occidentalis | lacm | 111708 | m | 1998 | Laramie Co., WY, USA |
| Bubo | virginianus | occidentalis | lacm | 21898 | f | 1916 | Kalevala, MB, CAN |
| Bubo | virginianus | occidentalis | lacm | 21899 | m | 1917 | Kalevala, MB, CAN |
| Melospiza | melodia | cooperi | lacm | 23558 | f | 1908 | Los Angeles, CA, USA |
| Melospiza | melodia | cooperi | lacm | 23556 | m | 1918 | Orange Co., CA, USA |
| Melospiza | melodia | cooperi | lacm | 23555 | m | 1905 | Los Angeles, CA, USA |
| Melospiza | melodia | cooperi | lacm | 23554 | m | 1905 | Los Angeles, CA, USA |
| Melospiza | melodia | cooperi | lacm | 19447 | f | 1915 | Los Angeles, CA, USA |
| Melospiza | melodia | cooperi | lacm | 17408 | m | 1930 | Los Angeles, CA, USA |
| Melospiza | melodia | saltonis | lacm | 3632 | f | 1919 | Riverside, CA, USA |
| Melospiza | melodia | saltonis | lacm | 3634 | m | 1919 | Riverside, CA, USA |
| Melospiza | melodia | saltonis | lacm | 3631 | m | 1919 | Riverside, CA, USA |
| Melospiza | melodia | saltonis | lacm | 3638 | f | 1919 | Riverside, CA, USA |
| Melospiza | melodia | saltonis | lacm | 3644 | m | 1919 | Riverside, CA, USA |
| Melospiza | melodia | saltonis | lacm | 3818 | f | 1919 | Riverside, CA, USA |
| Melospiza | melodia | caurina | lacm | 23519 | f | 1920 | POW Is., AK, USA |
| Melospiza | melodia | caurina | lacm | 23518 | m | 1920 | POW Is., AK, USA |
| Melospiza | melodia | caurina | lacm | 23517 | m | 1920 | POW Is., AK, USA |
| Melospiza | melodia | caurina | lacm | 23513 | f | 1920 | Grant Is., AK, USA |
| Melospiza | melodia | caurina | lacm | 23511 | m | 1920 | Wrangell, AK, USA |
| Melospiza | melodia | caurina | lacm | 23510 | f | 1920 | Wrangell, AK, USA |
| Melospiza | meloda | merrilli | lacm | 23465 | m | 1917 | Colusa Co., CA, USA |
| Melospiza | meloda | merrilli | lacm | 23466 | m | 1917 | Colusa Co., CA, USA |
| Melospiza | meloda | merrilli | lacm | 23467 | m | 1917 | Colusa Co., CA, USA |
| Melospiza | meloda | merrilli | lacm | 23468 | f | 1917 | Colusa Co., CA, USA |
| Melospiza | meloda | merrilli | lacm | 23469 | f | 1917 | Colusa Co., CA, USA |
| Melospiza | meloda | merrilli | lacm | 23470 | f | 1917 | Colusa Co., CA, USA |
| Corvus | corax | sinuatus | lacm | 120626 | m | 2013 | Los Angeles Co., CA, USA |
| Corvus | corax | sinuatus | lacm | 19566 | f | 1939 | San Clemente Is., CA, USA |
| Corvus | corax | sinuatus | lacm | 107319 | f | 1992 | Los Angeles Co., CA, USA |
| Corvus | corax | sinuatus | lacm | 35006 | f | 1958 | Isla Monserrate, Baja California, MX |
| Corvus | corax | sinuatus | lacm | 23894 | f | 1946 | Copala, Estado de Sinaloa, MX |
| Corvus | corax | sinuatus | lacm | 23893 | m | 1947 | Copala, Estado de Sinaloa, MX |
| Corvus | corax | principalis | lacm | 22475 | f | 1919 | POW Is., AK, USA |
| Corvus | corax | principalis | lacm | 22474 | f | 1919 | POW Is., AK, USA |
| Corvus | corax | principalis | lacm | 22473 | f | 1912 | Sitka, AK, USA |
| Cyanocitta | stelleri | stelleri | lacm | 21419 | f | 1919 | POW Is., AK, USA |
| Cyanocitta | stelleri | stelleri | lacm | 21418 | m | 1920 | POW Is., AK, USA |
| Cyanocitta | stelleri | stelleri | lacm | 21417 | f | 1920 | POW Is., AK, USA |
| Cyanocitta | stelleri | diademata | lacm | 29790 | f | 1950 | Barranca de Cobre, Chihuahua, MX |
| Cyanocitta | stelleri | diademata | lacm | 29789 | m | 1950 | Barranca de Cobre, Chihuahua, MX |
| Cyanocitta | stelleri | diademata | lacm | 29787 | m | 1950 | Cusaraga, Chihuahua, MX |
| Cyanocitta | stelleri | frontalis | lacm | 7321 | m | 1897 | San Bernardino, CA, USA |
| Cyanocitta | stelleri | frontalis | lacm | 21434 | m | 1917 | Colusa Co., CA, USA |
| Cyanocitta | stelleri | frontalis | lacm | 104979 | m | 1990 | Los Angeles Co., CA, USA |
