# Supplementary material for: Population- and Species-Level Variation in Near- and Mid-infrared Radiation in Birds: A Preliminary Analysis
Source: Integr Org Biol. 2026 Feb 28;8(1):obag006. doi: 10.1093/iob/obag006 (PMC13048275; doi:10.1093/iob/obag006)
Supplement: obag006_Supplemental_Files [file obag006_supplemental_files.zip › Supp Table 2.docx]

**Supplemental Table 2. Mean absorptance and emittance coefficients for each species.**

| Species (common name; abbreviation) | (normal-hemispherical) α - mean ± SD (range) | (normal-normal) α - mean ± SD (range) | ε - mean ± SD (range) |
| --- | --- | --- | --- |
| *B. virginianus*  (Great Horned Owl; owl) | N/A | 0.70 ± 0.05  (0.59 - 0.77)^a^ | 0.94 ± 0.02  (0.91 - 0.96)^a^ |
| *C. virginianus*  (Northern Bobwhite; bobwhite) | 0.81 ± 0.01  (0.80 - 0.83) | 0.73 ± 0.02  (0.68 - 0.76)^ab^ | 0.97 ± 0.005  (0.96 - 0.98)^b^ |
| *C. corax*  (Common Raven; raven) | N/A | 0.87 ± 0.02  (0.85 - 0.92)^c^ | 0.95 ± 0.01  (0.94 - 0.97)^a^ |
| *C. stelleri*  (Steller’s Jay; jay) | 0.83 ± 0.01  (0.81 - 0.85) | 0.77 ± 0.02  (0.74 - 0.79)^abc^ | 0.95 ± 0.004  (0.94 - 0.95)^a^ |
| *M. melodia*  (Song Sparrow; sparrow) | 0.82 ± 0.03  (0.75 - 0.85) | 0.80 ± 0.04  (0.73 - 0.83)^bc^ | 0.95 ± 0.008  (0.93 - 0.97)^a^ |

Letters indicate statistically significant differences across species within a column (p < 0.05).
