# Supplementary material for: Population- and Species-Level Variation in Near- and Mid-infrared Radiation in Birds: A Preliminary Analysis
Source: Integr Org Biol. 2026 Feb 28;8(1):obag006. doi: 10.1093/iob/obag006 (PMC13048275; doi:10.1093/iob/obag006)
Supplement: obag006_Supplemental_Files [file obag006_supplemental_files.zip › Supp Table 3.docx]

**Supplemental Table 3. Differences in bird-visible (300 - 700 nm) absorptance across populations.**

| Species | Subspecies* | Populations | α - mean ± SD (n) | P-value |
| --- | --- | --- | --- | --- |
| Owl** | *pallescens*  *pacificus*  *occidentalis* | California, USA  California, USA  Wyoming/Canada, USA | 0.84 ± 0.05 (3)  0.89 ± 0.008 (3)  0.87 ± 0.04 (3) | F = 1.923; df = 2; p = 0.23 |
| Bobwhite | *insignis*  *floridanus*  *mexicanus* | Chiapas, MX  Florida, USA  Iowa, USA | 0.97 ± 0.002 (3)  0.97 ± 0.006 (3)  0.97 ± 0.009 (3) | F = 0.02; df = 2; p = 0.98 |
| Raven** | *principalis*  *sinatus* (A)  *sinatus* (B) | Alaska, USA  California, USA  Sinaloa, MX | 0.98 ± 0.006 (3)  0.99 ± 0.01 (3)  0.99 ± 0.003 (3) | F = 0.49; df = 2; p = 0.63 |
| Jay | *stellari^a^*  *frontalis^b^*  *diademata^b^* | Alaska, USA  California, USA  Chihuahua, MX | 0.99 ± 0.004 (3)  0.97 ± 0.006 (3)  0.97 ± 0.003 (3) | F = 26.76; df = 2; **p = 0.001** |
| Sparrow | *caurina^a^*  *cooperi^ab^*  *merrilli^bc^*  *saltonis^c^* | Alaska, USA  S. California, USA (coastal)  N. California, USA  S. California, USA (desert) | 0.98 ± 0.008 (6)  0.98 ± 0.01 (6)  0.97 ± 0.003 (6)  0.95 ± 0.02 (6) | F = 13.18; df = 3; **p = 0.0005** |

*letters indicate statistically significant differences using Tukey’s or Dunnett’s T3 posthoc test (following ANOVA or Brown-Forsythe ANOVA).

**absorptance coefficients for owl and raven are normal-normal; the other species are normal-hemispherical.
