# Supplementary material for: Population- and Species-Level Variation in Near- and Mid-infrared Radiation in Birds: A Preliminary Analysis
Source: Integr Org Biol. 2026 Feb 28;8(1):obag006. doi: 10.1093/iob/obag006 (PMC13048275; doi:10.1093/iob/obag006)
Supplement: obag006_Supplemental_Files [file obag006_supplemental_files.zip › Supp Table 4.docx]

**Supplemental Table 4. Differences in average brilliance (400 - 700 nm) across populations.**

| Species | Subspecies* | Populations | Brilliance (%) - mean ± SD (n) | P-value |
| --- | --- | --- | --- | --- |
| Owl** | *pallescens*  *pacificus*  *occidentalis* | California, USA  California, USA  Wyoming/Canada, USA | 14.98 ± 4.44 (3)  9.81 ± 0.66 (3)  11.71 ± 3.47 (3) | F = 1.91; df = 2; p = 0.23 |
| Bobwhite | *insignis*  *floridanus*  *mexicanus* | Chiapas, MX  Florida, USA  Iowa, USA | 2.86 ± 0.18 (3)  2.81 ± 0.51 (3)  2.93 ± 0.80 (3) | F = 0.03; df = 2; p = 0.97 |
| Raven** | *principalis*  *sinatus* (A)  *sinatus* (B) | Alaska, USA  California, USA  Sinaloa, MX | 1.64 ± 0.64 (3)  1.08 ± 1.00 (3)  1.54 ± 0.31 (3) | F = 0.55; df = 2; p = 0.61 |
| Jay | *stellari^a^*  *frontalis^b^*  *diademata^b^* | Alaska, USA  California, USA  Chiahuahua, MX | 0.87 ± 0.40 (3)  2.70 ± 0.50 (3)  3.13 ± 0.30 (3) | F = 25.78; df = 2; **p = 0.0011** |
| Sparrow | *caurina^a^*  *cooperi^ab^*  *merrilli^b^*  *saltonis^b^* | Alaska, USA  S. California, USA (coastal)  N. California, USA  S. California, USA (desert) | 1.45 ± 0.94 (6)  2.17 ± 1.24 (6)  3.29 ± 0.26 (6)  4.95 ± 1.96 (6) | F = 8.87; df = 3; **p = 0.0027** |

*letters indicate statistically significant differences using Tukey’s or Dunnett’s T3 posthoc test (following ANOVA or Brown-Forsythe ANOVA).

**absorptance coefficients for owl and raven are normal-normal; the other species are normal-hemispherical.
