# Supplementary figures and images for: Population- and Species-Level Variation in Near- and Mid-infrared Radiation in Birds: A Preliminary Analysis
Source: Integr Org Biol. 2026 Feb 28;8(1):obag006. doi: 10.1093/iob/obag006 (PMC13048275; doi:10.1093/iob/obag006)

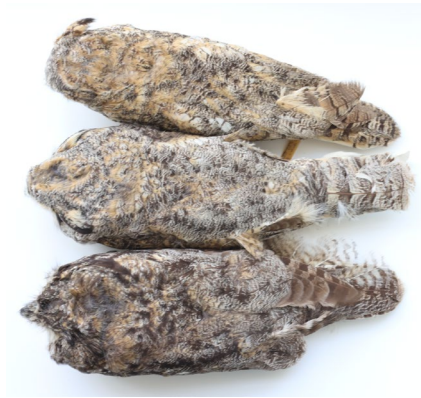

Great-horned Owl

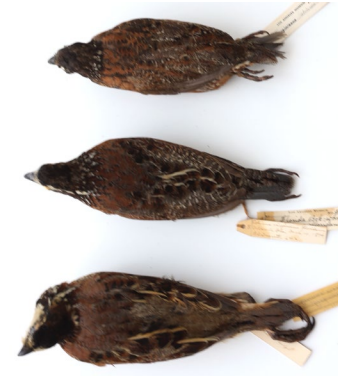

Northern Bobwhite

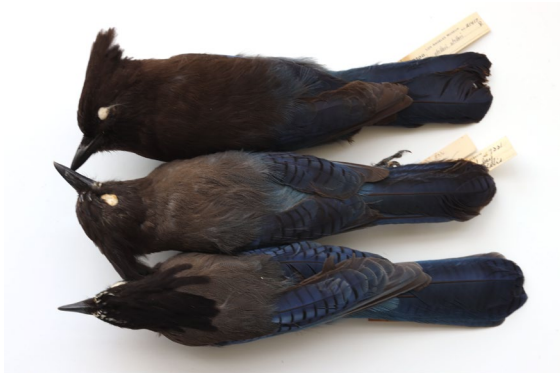

Steller's Jay

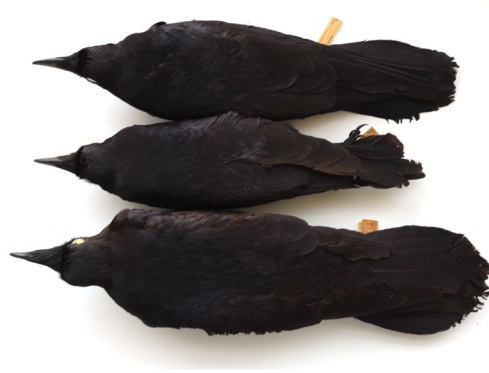

Common Raven

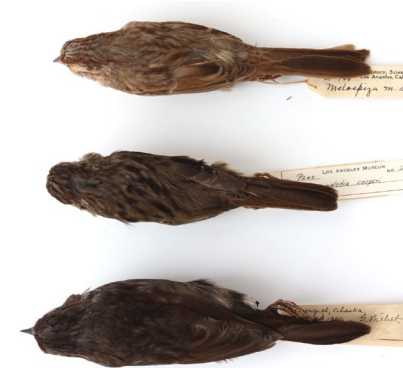

Song Sparrow

Supplement: obag006_Supplemental_Files [file obag006_supplemental_files.zip › SuppFig1.pdf]

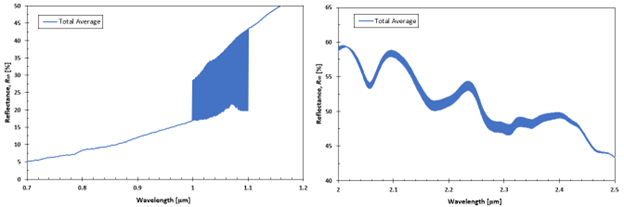

Supplement: obag006_Supplemental_Files [file obag006_supplemental_files.zip › SuppFig2.png]

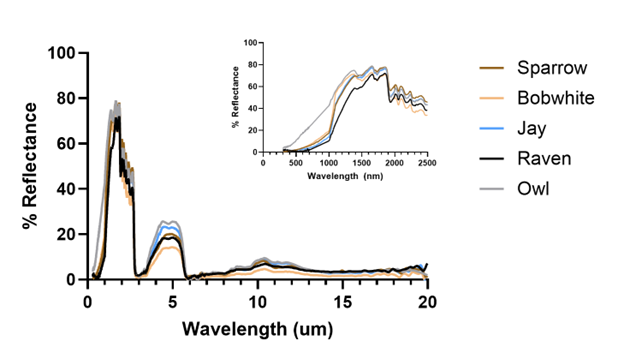

Supplement: obag006_Supplemental_Files [file obag006_supplemental_files.zip › SuppFig3.png]

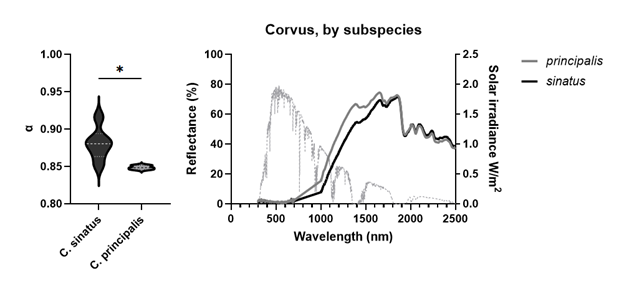

Supplement: obag006_Supplemental_Files [file obag006_supplemental_files.zip › SuppFig4.png]

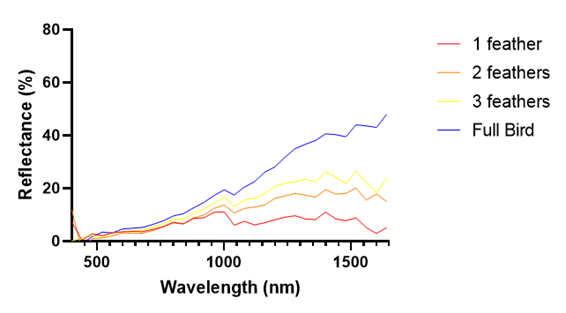

Supplement: obag006_Supplemental_Files [file obag006_supplemental_files.zip › SuppFig5.png]
